# Urine metabolomics for assessing fertility-sparing treatment efficacy in endometrial cancer: a non-invasive approach using ultra-performance liquid chromatography mass spectrometry

**DOI:** 10.1186/s12905-023-02730-4

**Published:** 2023-11-08

**Authors:** Junyu Chen, Jiale Liu, Dongyan Cao

**Affiliations:** 1 Department of Obstetrics and Gynecology, Qilu Hospital of Shandong University, Jinan, 250012 China; 2 Department of Obstetrics and Gynecology, Peking Union Medical College Hospital, National Clinical Research Center for Obstetric & Gynecologic Diseases, Chinese Academy of Medical Sciences & Peking Union Medical College, Beijing, 100730 China

**Keywords:** Endometrial cancer, Metabolomics, Fertility-sparing treatment, Diagnostic model

## Abstract

**Objective:**

This study aimed to reveal the urine metabolic change of endometrial cancer (EC) patients during fertility-sparing treatment and establish non-invasive predictive models to identify patients with complete remission (CR).

**Method:**

This study enrolled 20 EC patients prior to treatment (PT) and 22 patients with CR, aged 25–40 years. Eligibility criteria consisted of stage IA high-grade EC, lesions confined to endometrium, normal hepatic and renal function, normal urine test, no contraindication for fertility-sparing treatment and no prior therapy. Urine samples were analyzed using ultraperformance liquid chromatography mass spectrometry (UPLC-MS), a technique chosen for its high sensitivity and resolution, allows for rapid, accurate identification and quantification of metabolites, providing a comprehensive metabolic profile and facilitating the discovery of potential biomarkers. Analytical techniques were employed to determine distinct metabolites and altered metabolic pathways. The statistical analyses were performed using univariate and multivariate analyses, logistic regression and receiver operating characteristic (ROC) curves to discover and validate the potential biomarker models.

**Results:**

A total of 108 different urine metabolomes were identified between CR and PT groups. These metabolites were enriched in ascorbate and aldarate metabolism, one carbon pool by folate, and some amino acid metabolisms pathways. A panel consisting of Baicalin, 5beta-1,3,7 (11)-Eudesmatrien-8-one, Indolylacryloylglycine, Edulitine, and Physapubenolide were selected as biomarkers, which demonstrated the best predictive ability with the AUC values of 0.982/0.851 in training/10-fold-cross-validation group, achieving a sensitivity of 0.975 and specificity of 0.967, respectively.

**Conclusion:**

The urine metabolic analysis revealed the metabolic changes in EC patients during the fertility-sparing treatment. The predictive biomarkers present great potential diagnostic value in fertility-sparing treatments for EC patients, offering a less invasive means of monitoring treatment efficacy. Further research should explore the mechanistic underpinnings of these metabolic changes and validate the biomarker panel in larger, diverse populations due to the small sample size and single-institution nature of our study.

**Supplementary Information:**

The online version contains supplementary material available at 10.1186/s12905-023-02730-4.

## Introduction

Endometrial cancer (EC) is one of the most common gynecological cancers, with gradually rising incidence in recent years, especially among younger populations [1, 2]. About 15% of ECs occur in premenopausal women, and 5% are diagnosed in those of child-bearing age [3]. Consequently, fertility-preserving therapy has been applied in young EC patients with fertility aspirations [4]. Although fertility-sparing therapy offers a glimmer of hope for young women and has achieved a high response rate, literature reports indicate that the reproductive outcome remains poor, with only about 30% of patients becoming pregnant [5, 6]. Repeated hysteroscopic evaluation and diagnostic curettage during the treatment procedure may damage or cause adhesion to the endometrium, decreasing endometrial receptivity and lowering fertilized eggs implantation rates, which may cause the low rate of pregnancy [7]. Also, some drug of fertility-sparing treatment, such as Gonadotrophin releasinghormone agonist (GnRHa), can cause severe endometrial atrophy, complicating sampling and pathological diagnosis [8]. Besides, long-time use of drugs may cause side effect including obesity, abnormal liver function, thrombogenesis, osteoporosis, cardiovascular, and cerebrovascular diseases, it is crucial to identify biomarkers that can aid in evaluating treatment effectiveness and determining whether to continue the treatment [9]. Given the condition of low rate of pregnancy, limited understanding of treatment efficacy, the harm for invasive evaluations, and the absence of effective biomarkers for monitoring treatment response, a minimally invasive or noninvasive method with high specificity and sensitivity to evaluate the remission rate of fertility-sparing treatment, reduce the number of hysteroscopic operations, and improve the probability of pregnancy is required [10].

Metabolomics, as a minimally invasive or noninvasive emerging discipline, is focusing on small compounds with several major advantages, including relative ease of analysis, sensitivity to environment factors affecting pathogenesis and progression of disease, and minimal harm to the body [11–13]. It has been widely applied into various fields, including disease discovery, pharmacology, nutrition, toxicology and sport medicine [14, 15]. Previous research has identified differences in amino acids, lipids, and other metabolites between healthy women and EC patients [16–18]. Shao et al. found five urine diagnostic biomarkers, including porphobilinogen, acetylcysteine, N-acetylserine, urocanic acid and isobutyrylglycine which had great accuracy rate in discriminating 25 EC patients from 25 healthy controls [19]. Knific et al. built a diagnostic model between 65 EC patients and 61 controls using the ratio between acylcarnitine C16 and phosphatidylcholine PCae C40:1, the ratio between proline and tyrosine, and the ratio between the two phosphatidylcholines PCaa C42:0 and PCae C44:5, which provided sensitivity of 85.25%, specificity of 69.23%, and area under the curve (AUC) of 0.837 [18]. Cheng et al. identified biomarker of phosphocholine, asparagine, and malate with the AUC between 0.88 and 0.92 between 21 EC patients and 23 controls [20]. These metabolites have been proposed as biomarkers for diagnosing EC [21]. Although metabolomics has started to shed light on disease mechanisms in EC, no previous studies tackled the evaluation of fertility sparing treatment in EC patients using metabolomics.

Ultra-Performance Liquid Chromatography-Mass Spectrometry (UPLC-MS) is a highly sensitive and specific analytical method that combines the separation capabilities of UPLC with the qualitative and quantitative analysis capabilities of MS [22]. It is particularly useful for metabolic profiling and biomarker discovery, allowing for the identification and quantification of metabolites present in urine samples [23, 24]. This technique enables us to explore the metabolic changes in EC patients undergoing fertility-sparing treatment, and identify potential specific biomarkers for evaluating conservative treatment effectiveness, thereby providing new insights into treatment effectiveness.

Since many metabolites are excreted through the kidney, metabolites are often more concentrated in urine than in blood, providing a robust and sensitive medium for detection [25]. In addition, early small changes in blood are eliminated due to homeostatic mechanisms, while urine collects waste from the entire body and exhibits more abundant changes [26]. Moreover, urine samples are easier to collect, store, and analyze compared to blood samples. Therefore, urine can better reflect the body’s metabolic state at early-stage of the disease and is expected to become an essential method for screening biomarkers [27]. In light of these advantages, our study employed UPLC-MS metabolomics to analyze urine samples as a sensitive and relevant tool for evaluating the effectiveness of fertility-sparing treatments in patients with EC.

In summary, our objective is to utilize urine metabolomics to reveal metabolic changes in EC patients undergoing fertility-preserving treatment, thereby providing a novel, non-invasive method for treatment evaluation and enhancing our understanding of underlying metabolic mechanisms.

## Materials and methods

In this observational study, patients were recruited from April 2020 to June 2021 at the Department of Obstetrics and Gynecology, Peking Union Medical College Hospital (PUMCH). Patients’ information such as age, height, weight, laboratory indices (including blood and urine routine examination, measures of liver and kidney function, level of tumor marker, and other relevant biochemical measures), treatment response, and other information were obtained from the medical and laboratory reports.

### Eligibility criteria

#### Inclusion criteria

(1) Histologically confirmed EC, G1; (2) Women aged 18–40 years who have strong desire to preserve their uterus; (3) The lesion was confined to the endometrium confirmed by imaging study; (4) Normal hepatic functions (ALT, AST, etc.), renal functions (Cr, BUN, etc.) and urine test (UWBC, URBC, urine protein, etc.); (5) No contraindication for fertility-sparing treatment; (6) No prior therapy received by patients; (7) All participants provided written informed consent.

#### Exclusion criteria

(1) Patients with diseases potentially affect metabolism, such as thyroid dysfunction (hyperthyroidism or hypothyroidism), and immunodeficiency diseases; (2) Patients with other forms of cancer; (3) Patients received any form of cancer therapy, such as radiation and chemotherapy.

### Patients classification

All patients received the same fertility-sparing treatment regimen: 500 mg daily of oral medroxyprogesterone acetate (MPA). The typical duration of treatment varied based on individual response, but generally spanned between 3 and 6 months. During treatment, patients underwent endometrial curettage under hysteroscopic evaluation every three months to monitor the response. Patients were divided into pre-treatment (PT) group and the complete remission (CR) group according to the pathological results.

PT Group: Patients who had been diagnosed with EC but had not yet initiated any form of fertility-sparing treatment at the time of urine sample collection.

CR Group: Patients who, following fertility-sparing treatment, exhibited no evidence of EC verified through pathology [28].

A total of 42 women were included in this study, including 22 PT patients and 20 CR patients.

### Urine sample collection and preparation

Midstream urine samples were collected from all participating patients in sterile, single-use containers. The samples were obtained before hysteroscopic evaluation after sterilizing vulva and vagina to minimize contamination. Upon collection, the samples were immediately stored on ice and transferred to the laboratory within 1 h. The urine samples were stored at − 80 °C before analysis. In the lab, 200 µl urine sample was mixed with 200 µl acetonitrile and swirled for 30 s. Subsequently, the sample was subjected to centrifugation at 14,000×g for 10 min to precipitate solid impurities. The resulting supernatant was carefully decanted, vacuum-dried to a fine powder form, and stored at a temperature of -40 °C until further analysis to preserve the integrity of the metabolites. Before undergoing UPLC-MS analysis, the dry powder was redissolved in 100 µl of 2% acetonitrile. To eliminate small-protein interference, a 10 kDa molecular weight cutoff ultracentrifugation filters was used prior to transferring the samples to an autosampler for analysis.

### UPLC-MS analysis

Waters ACQUITY H-class LC system coupled with a Triple TOF 5600 mass spectrometer were applied to UPLC-MS analyses of urine samples. Metabolites were separated with a 15-minute gradient on a Waters HSS C18 column (3.0 × 100 mm, 1.7 μm) at a flow rate of 0.5 mL/min. Mobile phase A consisted of 0.2‰ formic acid in H_2_O, and mobile phase B was 0.2‰ formic acid acetonitrile solution. The gradient was set as follows: 0–1 min, 2% solvent B; 1–3 min, 2–15% solvent B; 3–6 min, 15–50% solvent B; 10–10.1 min, 95% solvent B; 10.1–12 min, 95–2% solvent B; 12–15 min, 2% solvent B. The column temperature was set at 40◦C. The eluted metabolites were analyzed by Triple TOF 5600 mass spectrometers, with data collection in DDA mode. Parameters were as follows: First-level full scan range: 50-1200 m/z, cumulative time: 0.25s, second-level cumulative time:0.1s, GS1: 55, GS2: 55, Curtain Gas: 35, temperature: 550℃, Ionspray Voltage Floating: 4500 V.

### Quality control

The quality control (QC) samples, prepared by mixing equal aliquots from each individual biological sample, serve as internal standards to monitor the performance and stability of the instrument over time. The QC samples were injected between every ten samples to assess the stability and repeatability of the analytical process. In total, five QC injections were carried out throughout the entire analysis, serving as checkpoints for data quality and instrument performance.

### Data processing

Data acquisition from UPLC-MS was subjected to rigorous pre-processing and statistical analyses to ensure the validity and robustness of our results. Raw data files were initially processed using Progenesis QI software following previously established strategies [29, 30]. Data were imported into MetaboAnalyst 5.0 (http://www.metaboanalyst.ca) for further processing. Data normalization, log transformation and quality control sample correction were applied during the process.

#### Filling missing values

Variables missing more than 50% of the samples were discarded to minimize the influence of outliers. Additionally, variables with a coefficient of variation (CV) greater than 0.5 were also excluded to ensure that only reliably quantifiable metabolites were considered. In our dataset, less than 5% missing data, 2015 missing values among 40,764 values, were observed. We utilized the K-nearest Neighbor algorithm to impute missing values within each group to maintain the integrity of the dataset (set k to 5). Additionally, we used the minimum value method for filling in missing values between the CR and PT groups to provide a conservative estimate and avoid inflating the significance of our findings.

#### Pattern recognition and significant metabolites identification

Data were analyzed using pattern-recognition methods, including principal component analysis (PCA) and orthogonal partial least squares discriminant analysis (OPLS-DA), with the software Simca version 14.1 [31, 32]. Metabolites with a p-value < 0.05, Variable Importance in the Projection (VIP) >1, and Fold-change > 1.5 were considered significant differential metabolites. These cutoffs were chosen based on their common usage in metabolomics studies and their suitability for minimizing false positives while maximizing true discoveries.

#### Validation and prediction accuracy

The MetaboAnalyst 5.0 platform was used to conduct a receiver operating characteristic (ROC) analysis, a graphical method used for evaluating the ability of a binary, and 10-fold cross-validation, a technique used to assess how well a predictive model will generalize to an independent dataset, to assess the predictive accuracy of the model, providing an accurate measure of the model’s discriminatory capability [33–36].

## Results

A total of 42 women were included in this study, including 22 PT patients and 20 CR patients. The demographic and clinical characteristics of the study are summarized in Table 1 (Additional details in Supplementary Table S1). Groups were matched by age, height, weight, and body mass index (BMI), and the pathological diagnosis of each patient was confirmed by two professional pathologists after hysteroscopic evaluation. The liver and renal function, along with other laboratory indices, were within the normal range and also matched in each group. No statistically significant differences were found in each subgroup, except for the level of CA125. Patients with CR have a lower level of CA125 compared to patients with PT (p = 0.0016, Mann-Whitney U test).

**Table 1:** The baseline information of enrolled subjects in the study

Characteristics	PT (n = 22)	CR(n = 20)
Age (years), median (range)	35 (32–37)	32 (30–34)
BMI (kg/m^2^), median (range)	28.2 (24.4–30.9)	26.8 (25.4–31.8)
Laboratory examination, median (range)		
ALT (U/L)	19 (11–38)	17 (8–33)
Cr (µmol/L)	57 (44–74)	62 (33–76)
Glucose (mmol/L)	5.3 (4.6–12.1)	5.1 (4.0–7.0)
TC (mmol/L)	5.2 (3.3–6.6)	5.1 (3.72–6.2)
TG (mmol/L)	1.1 (0.3–2.8)	1.1 (0.4–3.2)
HDL (mmol/L)	1.3 (0.7–3.7)	1.1 (0.8–2.2)
LDL (mmol/L)	3.0 (1.6–4.8)	3.0 (1.6–4.6)
Urine WBC	0-Trace	0-Trace
Urine RBC	0-Trace	0-Trace
Urine protein (g/L)	0	0
Urine glucose (mmol/L)	0	0
CA125 (U/mL)	20.1 (10.5–32.8)	13.7 (5.5–31.3)
Comorbidity (n, %)		
DM	3 (13.6%)	3 (15.0%)
HP	2 (9.1%)	2 (10%)
PCOS	2 (9.1%)	2 (10%)

Notes: BMI = body mass index, DM = diabetes mellitus, HP = hypertension, PCOS = polycystic ovary syndrome.

**Fig. 1:**
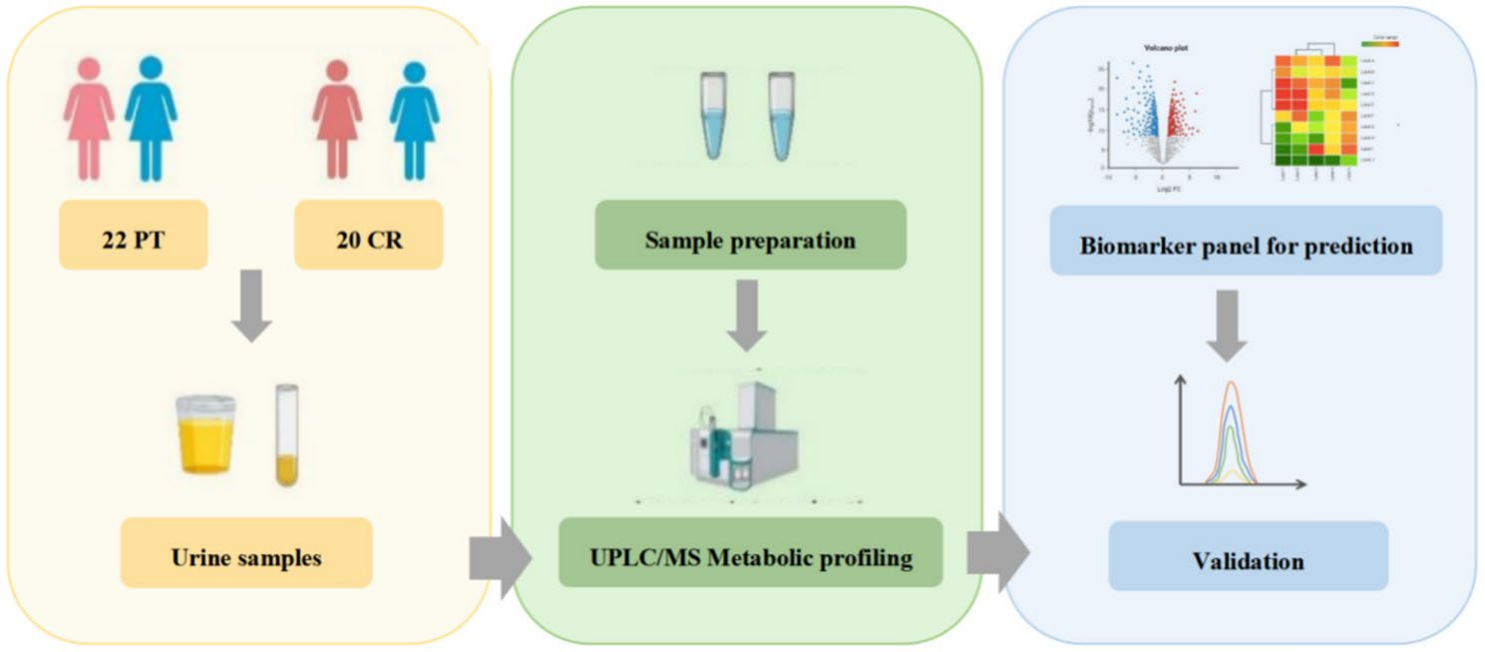
The workflow of this study

A differential analysis on urine metabolomics was performed to discriminate CR patients from PT patients. Biomarker panels were discovered based on metabolic profiling analysis. The workflow of the study, outlining the steps of sample collection, metabolic profiling, and data analysis, is presented in Fig. 1. The QC sample clustering, shown in Figure S1, demonstrates the reliability of the metabolomics analyses (p < 0.05, t-test for variance). After removal of the missing values more than 50% of samples, values with CV > 0.5, score < 40, and fragmentation < 20, a total of 947 urine features were selected for further analysis.

**Fig. 2:**
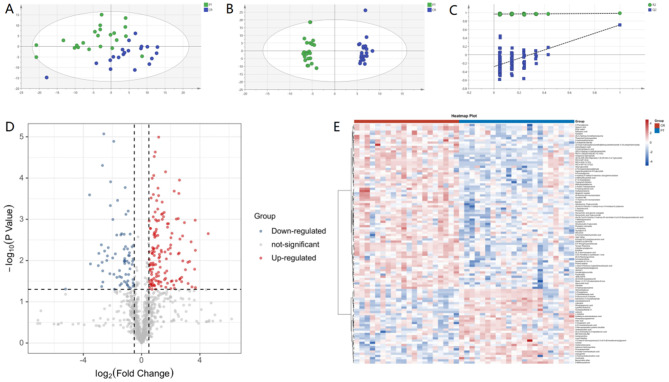
Metabolic analysis between pre-treatment and complete remission patients. **A.** PCA score plot of urine metabolome; **B** OPLS-DA score plot of urine metabolome; **C**. 100 permutation test of the OPLS-DA model in samples; **D**. Volcano plot of differential metabolites between the two groups; E. Heatmap of differential metabolites between the two groups

The differences between these two groups could be observed from the PCA score plot (Fig. 2A), and the OPLS-DA model achieved a better separation (Fig. 2B). One hundred permutation tests demonstrated the stability and robustness of the supervised models (Fig. 2C).

**Fig. 3:**
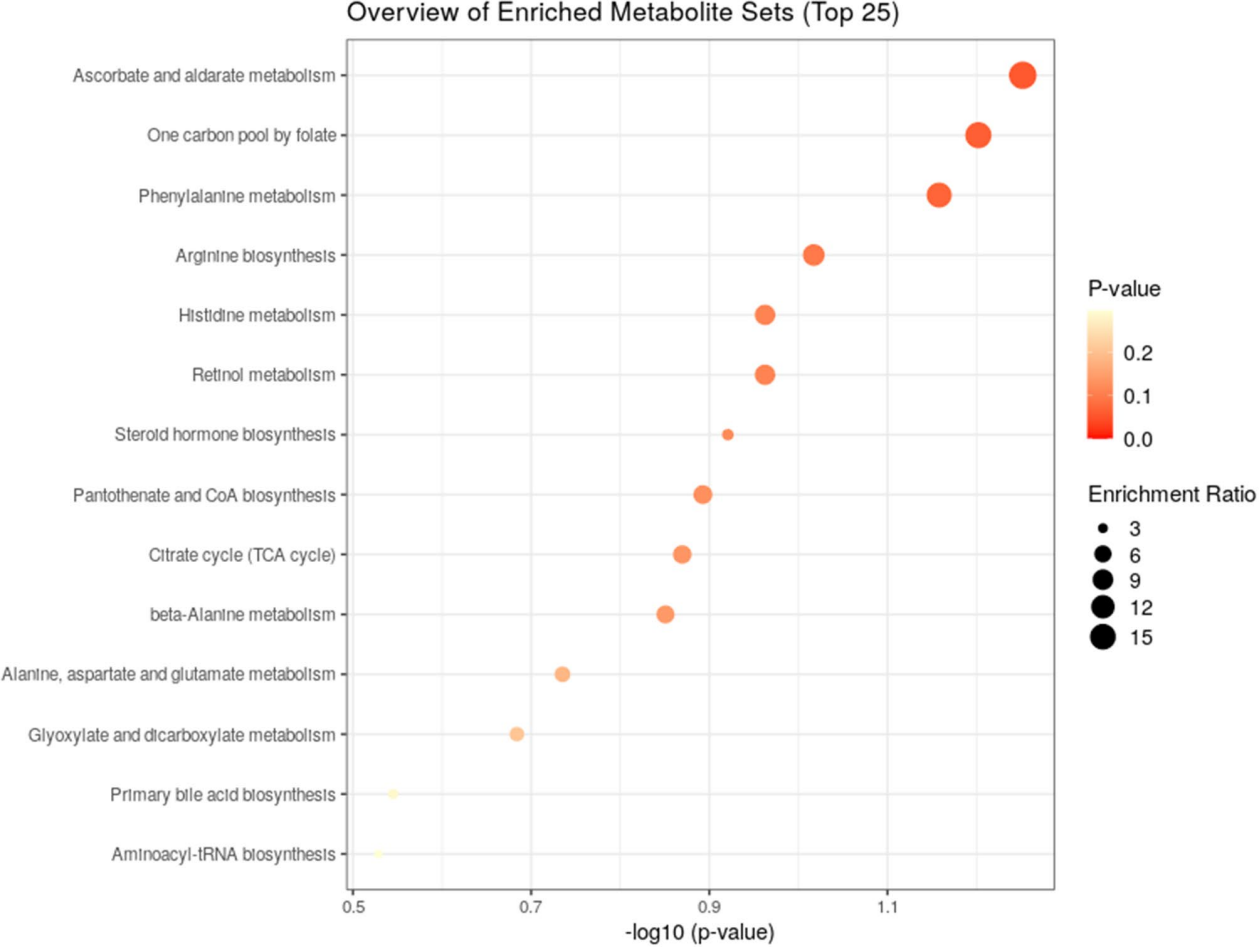
Pathway enrichment analysis related to differential urine metabolites between pre-treatment and complete remission patients

In total, 108 differential metabolites were identified (Fig. 2D and E), which were further submitted for pathway analysis and prediction model construction. Pathway enrichment analysis showed enrichment in ascorbate and aldarate metabolism, one carbon pool by folate, phenylalanine metabolism, arginine biosynthesis, histine metabolism, etc. (Fig. 3) [37–39].

**Fig. 4:**
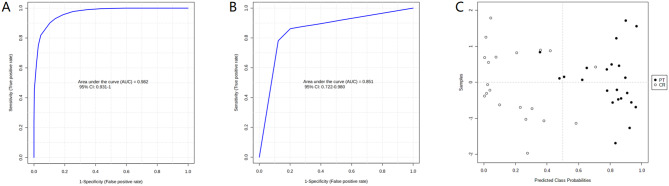
ROC curves for the proposed diagnostic model. ROC curve based on urine biomarker panel in discovery group; **B.** ROC curve of 10-fold cross-validation of the biomarker panel; **C.** Predicted probability plot of metabolite panel

The diagnostic accuracy of identified differential metabolites was evaluated (Table S2). A total of 32 metabolites demonstrated potential diagnostic ability with an AUC above 0.8, and 2 metabolites exhibited an AUC above 0.9. A multivariate ROC curve-based exploratory analysis was performed to achieve a better predictive model using a logistic regression algorithm. The panel consisting of Baicalin, 5beta-1,3,7 (11)-Eudesmatrien-8-one, Indolylacryloylglycine, Edulitine, and Physapubenolide exhibited the best predictive ability (Table 2). The AUC value of the panel was 0.982 (0.931 ~ 1.000) for the discovery group with a sensitivity of 0.975 and a specificity of 0.967, which indicated that the biomarker panel is highly effective in differentiating between the CR and PT groups in the discovery cohort. For 10-fold cross-validation, the AUC value was 0.851 (0.722 ~ 0.980) (Fig. 4), although lower than the AUC for the discovery group, still indicates strong predictive power.

**Table 2:** Prediction ability of metabolite panel for complete remission

Parameter		AUC	Sensitivity	Specificity
^a^Urine metabolites panel	Training/Discovery	0.982 (0.931 ~ 1.000)	0.975 (0.953 ~ 0.997)	0.967 (0.940 ~ 0.993)
	10-fold Cross-Validation	0.851 (0.722 ~ 0.980)	0.864 (0.864 ~ 1.000)	0.900 (0.769 ~ 1.000)

^a^The urine biomarker panel consisting of Baicalin, 5beta-1,3,7 (11)-Eudesmatrien-8-one, Indolylacryloylglycine, Edulitine, and Physapubenolide

## Discussion

For patients receiving fertility preserving therapy, the tumor burden decreases as the disease gradual remits, and the metabolic pathways change accordingly. Thus, predicting the degree of disease remission can be achieved by comparing the changes of metabolites in CR and PT patients using metabolomics. In this study, we conducted a UPLC-MS-based metabolomics analysis to reveal the metabolic changes in EC patients during fertility-sparing treatment and establish diagnostic models to evaluate the treatment effect, which offer a broader scope to better capture the circulating metabolic features of EC patients receiving fertility-sparing treatment.

In the comparison between patients with CR and PT, the differential metabolites were mainly enriched in the ascorbate and aldarate metabolism. This specific metabolic pathway plays an important role in managing oxidative stress, which is closely associated with various pathologies and disorders, such as cardiovascular disease, aging, neurodegenerative diseases, and cancers [40–42]. Also, high-doses of ascorbate can induce pro-oxidative effects and selectively kill cancer cells, although the mechanism of action is not fully understood [43, 44]. This indicates that a shift in this metabolic pathway could be instrumental in the treatment of EC. However, to our knowledge, changes of ascorbate and aldarate metabolism has not previously been linked with fertility-sparing treatment of EC. Our observations corroborate existing research on the anomalies in ascorbate and aldarate metabolism in various cancers [45–47]. The changes of this pathway in our study may be due to the inhibition of energy metabolism of EC cancer cells during the disease remission. Therefore, this metabolic pathway and related genes could serve as potential therapeutic targets for the fertility-sparing treatment of EC. In comparison to existing literature on the subject, our findings add new dimensions to the understanding of metabolic changes in patients undergoing fertility-sparing treatment for EC. While previous studies have examined changes in specific metabolic pathways in relation to various cancers, our study is the first to link alterations in ascorbate and aldarate metabolism with fertility-sparing treatments in EC.

Another crucial metabolic pathway displaying differences between the two groups was folic acid metabolism. Folic acid, a primary supplier of single-carbon chains, serves as a cofactor for the initial synthesis of purines and thymidines, which plays a key role in maintaining the genetic and epigenetic stability of DNA, and is directly involved in the organism’s growth, development or reproduction processes [48]. Folic acid deficiency impairs the conversion of nucleic acid to deoxythymine monophosphate, essential for DNA synthesis and repair. Misbinding of uracil to thymine leads to DNA instability, DNA strand breaks, DNA repair errors, and altered methylation status on a genomic scale [49]. Consequently, folic acid deficiency is linked to various cancers, including head and neck cancers, nasopharyngeal cancers, esophageal cancers, pancreatic cancers, bladder cancers and cervical cancers [50–52]. In this study, folic acid levels were significantly elevated in patients with CR, consistent with previous research. Based on the observed increase in folic acid levels in patients with CR, we hypothesized that higher folic acid intake could potentially have a therapeutic effect in reversing EC. But this interpretation requires further study for validation. However, excessive folic acid could promote the growth and progression of existing tumors, negatively correlating with EC development [53]. Besides, another report indicated that dietary levels of folic acid do not seem to affect the incidence of EC [54]. Therefore, further well-designed prospective studies or randomized clinical trials are necessary to investigate the folic acid’s effect on EC. Our findings are partially consistent with earlier studies and provide new insights into its potential significance in fertility-sparing treatments, which warrants further investigation.

Metabolic pathways are not isolated entities but are part of a complex network that regulates cellular homeostasis. Ascorbate metabolism plays an essential role in scavenging reactive oxygen species (ROS) and maintaining the redox status within cells [41]. Elevated ROS levels can lead to oxidative stress, which has been linked to DNA damage, aberrant cell proliferation, and tumorigenesis [55]. Folic acid metabolism is vital for the synthesis of purines and pyrimidines, the building blocks of DNA. Disruption in folic acid metabolism can affect DNA replication and repair, making cells susceptible to genetic mutations and tumorigenic transformations [56, 57]. In the context of EC, the interplay between ascorbate and folic acid metabolism could be of particular significance. The balance between these pathways might be essential for the efficacy of fertility-sparing treatments. For instance, optimizing ascorbate levels could mitigate oxidative stress-induced damage, while ensuring proper folic acid metabolism might support DNA integrity during cell division. The disruptions in one pathway could affect the other, resulting in a cascade of metabolic imbalances that could either promote or inhibit tumor progression. While our study provides a foundational understanding of these metabolic alterations in EC, it also raises some questions about the deeper mechanistic interplay between these and other metabolic pathways, such as how other cofactors involved in DNA synthesis and repair interact with ascorbate and folic acid metanolism, and are there feedback loops or regulatory checkpoints that modulate these pathways. Further studies are warranted to unravel the complexities of these interactions, providing more comprehensive insights into the metabolic landscape of EC. Such findings could pave the way for novel therapeutic strategies, targeting the metabolic vulnerabilities of endometrial cancer cells to enhance the efficacy of fertility-sparing treatments.

In the diagnostic model, Baicalein, found in the roots of Scutellaria baicalensis and Scutellaria lateriflora, has been reported to exhibit anticancer activity against various cancers, including pancreatic, prostate, lung, breast, liver, gastric and colon cancers [58–63]. It targets multiple sites and employs diverse pathways to induce apoptosis or programmed cell death [64]. In our study, Baicalein levels were found to be increased in CR patients, suggesting its potential as a tumor marker. Furthermore, numerous studies have demonstrated that baicalein enhances the efficacy of certain drugs potentially used in chemoprevention and anti-cancer therapy, indicating that it could also serve as potential drug for fertility-sparing treatment in patients [65–67]. However, it is crucial to acknowledge that while our findings suggest potential therapeutic implications of Baicalein, its clinical utility and safety in the specific context of EC and fertility-sparing treatment remain uncertain. More extensive studies and clinical trials are needed to further explore its efficacy and safety profile in this specific patient population.

### Limitations

While our study sheds new light on the metabolic changes associated with fertility-sparing treatment in EC, several limitations should be noted. Firstly, the sample size of our study was relatively small, which may limit the generalizability of our findings. Secondly, the study was conducted at a single institution, which may introduce bias and reduce the diversity of patient populations examined. Thirdly, although the groups were matched by age, height, weight, and BMI, other uncontrolled variables like dietary habits, and physical activity could also impact metabolic pathways and thus represent confounding factors. Finally, ethical responsibilities, and data confidentiality also demand conscientious consideration, ensuring the validated, ethical application of the findings in treatment decisions.

## Conclusion

This study performed urine metabolomics approach to investigate the metabolic features of EC patients with fertility-sparing treatment, which was approved by the Ethics Committee of PUMCH (ZS-2666). The results reveal markedly different metabolic profiles between patients with CR and PT groups, suggesting the feasibility of using metabolites for effect evaluation and provide new insights into the pathogenesis of diseases and potential targets for fertility-sparing treatment. Potential biomarkers were also explored and proved to have significant diagnostic value, which could help determine the appropriate time to terminate treatment, reduce the number of operations, and minimize endometrial damage. However, our study’s limitations call for larger, multi-center studies to validate our preliminary results, and future investigations should ensure rigorous ethical oversight throughout their studies.

### Electronic supplementary material

Below is the link to the electronic supplementary material.

Supplementary Material 1

Supplementary Material 2

Supplementary Material 3

## Data Availability

Data and materials are available from the corresponding author by reasonable request.
