# Supplementary material for: Urine metabolomics for assessing fertility-sparing treatment efficacy in endometrial cancer: a non-invasive approach using ultra-performance liquid chromatography mass spectrometry
Source: BMC Womens Health. 2023 Nov 8;23:583. doi: 10.1186/s12905-023-02730-4 (PMC10634093; doi:10.1186/s12905-023-02730-4)
Supplement: Supplementary file 3 — Supplementary Material 3 [file 12905_2023_2730_MOESM3_ESM.docx]

**Fig. S1 UPLC-MS system stability assessment.**

Yellow, samples; Red, QCs.


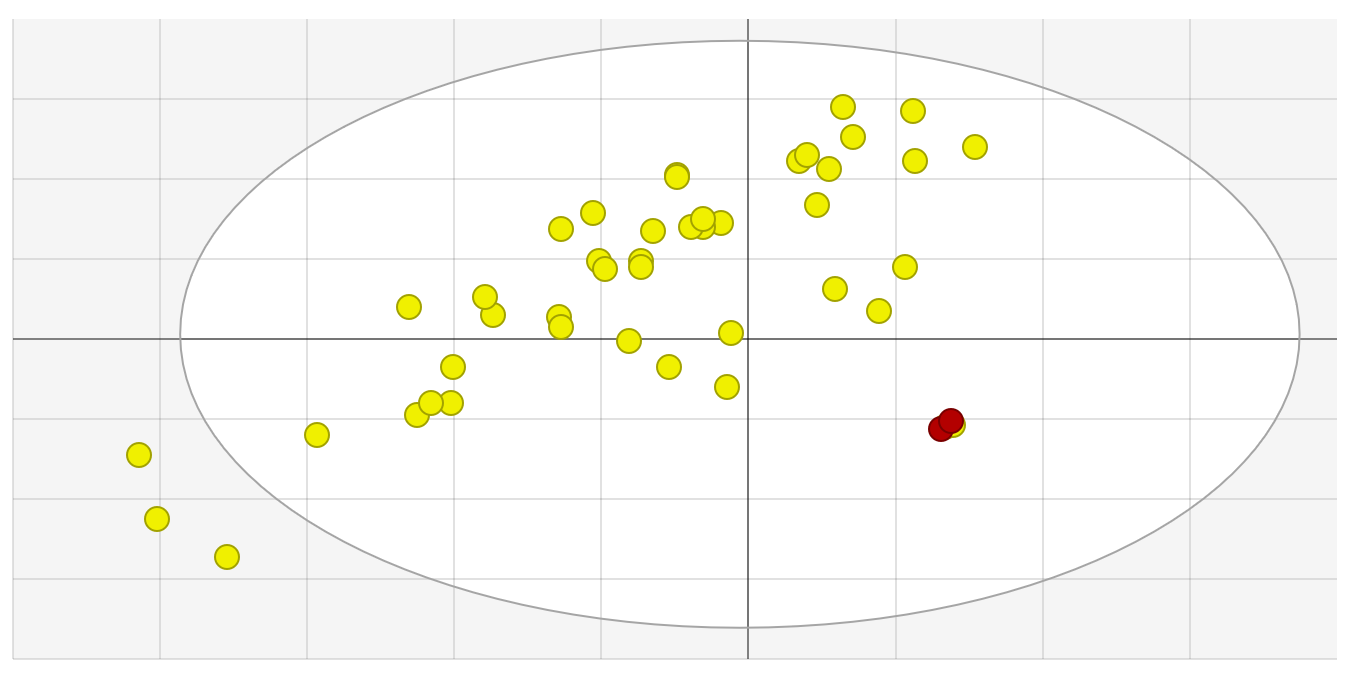


**Fig. S2 Box plots for metabolites consisting of biomarker panel**

A. Box plot for Baicalin; B. Box plot for 5beta-1,3,7(11)-Eudesmatrien-8-one; C. Box plot for Indolylacryloylglycine; D. Box plot for Edulitine; E. Box plot for Physapubenolide.

**
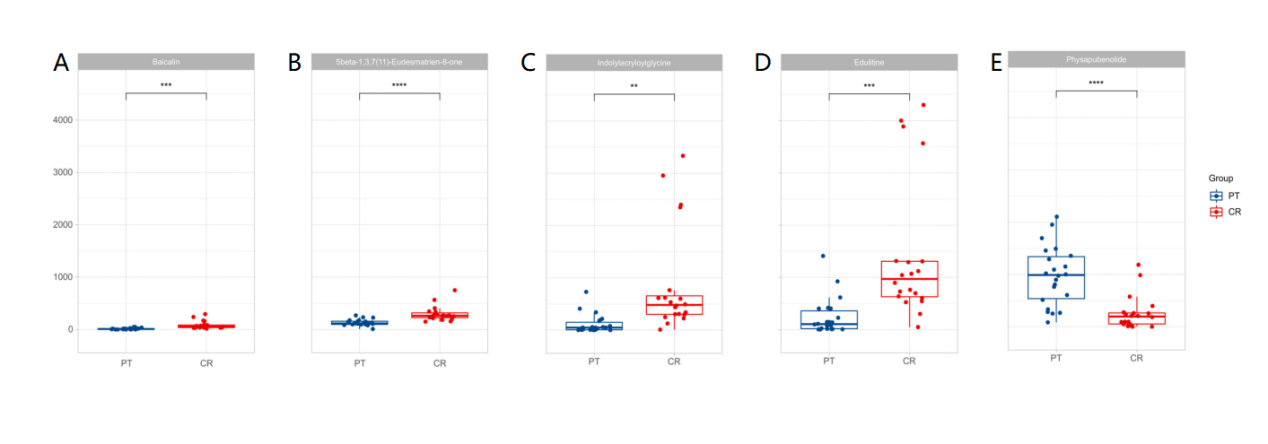
**
